# One-step RT-droplet digital PCR: a breakthrough in the quantification of waterborne RNA viruses

**DOI:** 10.1007/s00216-013-7476-y

**Published:** 2013-11-26

**Authors:** Nejc Rački, Dany Morisset, Ion Gutierrez-Aguirre, Maja Ravnikar

**Affiliations:** 1 Department of Biotechnology and Systems Biology, National Institute of Biology, Vecna pot 111, 1000 Ljubljana, Slovenia; 2 Centre of Excellence for Biosensors, Instrumentation and Process Control-COBIK, Velika pot 22, 5250 Solkan, Slovenia

**Keywords:** Waterborne virus, Quantification, Droplet digital PCR, Real-time PCR, Digital PCR

## Abstract

Water contamination by viruses has an increasing worldwide impact on human health, and has led to requirements for accurate and quantitative molecular tools. Here, we report the first one-step reverse-transcription droplet digital PCR-based absolute quantification of a RNA virus (rotavirus) in different types of surface water samples. This quantification method proved to be more precise and more tolerant to inhibitory substances than the benchmarking reverse-transcription real-time PCR (RT-qPCR), and needs no standard curve. This new tool is fully amenable for the quantification of viruses in the particularly low concentrations usually found in water samples.

**Electronic supplementary material:**

The online version of this article (doi:10.1007/s00216-013-7476-y) contains supplementary material, which is available to authorized users.

## Introduction

Enteric viruses in drinking water and in ground and recreational water systems are responsible for the large majority of waterborne disease outbreaks [1, 2]. Enteric viruses are found in the host gut, tears and aerosols and are excreted with the feces at concentrations of up to 10^13^ virus particles per gram of stool [3], leading to the release of large amounts of viruses into raw sewage and environmental water sources [4]. Recent outbreaks of enteric viruses in Finland [5] and Montenegro [6] confirm the need for immediate action in waterborne virus diagnostics. Detection and quantification of these viruses in such environments is especially challenging due to their low titer and the presence of inhibitory substances. With an infectious dose as low as ten virus particles [7], very sensitive detection tools are required. Regulatory decisions regarding microbial water quality are being shifted towards the use of quantitative risk based approaches (QMRA—quantitative microbiological risk assessment) [8] leading to a growing need for accurate and absolute quantification of waterborne pathogens [8, 9].

Real-time PCR (qPCR), enables detection and quantification of target nucleotide sequences down to a few copies and is, at present, the method of choice in the field of water quality determination [9]. However, it can be influenced by inhibitory substances found in environmental waters, which affect the accuracy of viral quantification [9].

Recently, digital PCR (dPCR) has gained increasing popularity [10] due to its upgrading to the so-called “droplet digital PCR” (ddPCR) systems which, compared to other platforms of dPCR, enable a significant gain in dynamic range while decreasing the cost of analysis [10, 11]. In dPCR, the reaction mix is distributed across a large number of partitions containing zero, one or more copies of the target nucleic acid. After endpoint PCR amplification, each partition is scrutinized and defined as positive (“1”, presence of PCR product) or negative (“0”, absence of PCR product) hence the term “digital”. The absolute number of target nucleic acid molecules contained in the original sample before partitioning can be calculated directly from the ratio of positive to total partitions, using binomial Poisson statistics [11].

Unlike qPCR quantification, which is based on the use of a standard curve, ddPCR is an endpoint and absolute measurement approach that enables the determination of target copy number without the need of a standard. It therefore avoids the amplification efficiency bias, due to matrix linked inhibition, observed with qPCR [12, 13]. It is more sensitive than qPCR for rare targets [14] and provides more accurate data of metrological quality [12, 13], especially at low target copy numbers [15–17]. Moreover, as an endpoint measurement, the signal in ddPCR is measured only after finishing the PCR amplification. This can reduce the biases linked to matrix type components (inhibitors) often observed with qPCR. Quantification in ddPCR is done upon presence or absence of signal and not upon changes in intensity of fluorescence (as in qPCR) therefore quantification is less affected by the shift of intensity of fluorescence that some inhibitors can induce [17, 18]. Only a few DNA targets (including DNA viruses) [19] have so far been quantified using ddPCR and there have been no reports of its application to viral RNA targets. Recently, guidelines about the minimum information to be provided for publication of quantitative digital PCR experiments have been published [20].

The aim of this study was therefore to evaluate the potential of ddPCR for accurate quantification of a waterborne RNA virus at low concentrations in a one-step format. To confirm such proof of principle, we used a well characterized, rotavirus specific, reverse-transcription (RT) qPCR assay [21] and adapted it directly to a one-step RT-ddPCR assay, without modification. RT-ddPCR performance, using a commercialized droplet digital PCR platform, was compared with the benchmark RT-qPCR. Linearity of response, absolute limits of detection and quantification, repeatability over the dynamic range of the RT-ddPCR assay were assessed. The applicability of ddPCR was also evaluated on different environmental water samples. This study opens up a new concept of quantification for other RNA viruses and applications.

## Materials and methods

### Virus and water samples

A rotavirus (RoV) clarified suspension was derived from routine rotavirus positive clinical stool samples collected at the Institute for Microbiology and Immunology, University of Ljubljana, Slovenia. Virus concentration was estimated using electron microscopy and latex particle counting with a JEM 1200 EXII instrument (Jeol, Tokyo, Japan) at the same institute. The estimated rotavirus concentration was 1.1 × 10^11^ particles/ml. This suspension was used as an inoculum in all experiments.

The effluent sample was obtained from a local wastewater treatment plant (Central Waste Water Treatment Plant Domžale-Kamnik, Ihan, Slovenia). The environmental samples were collected from different sources of surface waters within the Ljubljana metropolitan area (Slovenia). Physico-chemical parameters of each environmental sample were determined: pH was measured using SevenMulti pH meter (Mettler Toledo, Switzerland), conductivity with Multiline P4, Multi Measuring device (WTW, Germany) and turbidity using HI 93703 Portable Microprocessor Turbidity Meter (Hanna Instruments, Portugal). After collection, samples were stored at 4 °C until processing.

### Dilutions

For dynamic range determination, the initial RoV suspension (1.1 × 10^11^ rotavirus particles/ml) was first diluted to 10^10^ rotavirus particles/ml, in milliQ water (ultrapure milliQ water, EMD Millipore MA, USA). From this concentration further tenfold serial dilutions were prepared all the way to 10^0^ rotavirus particles/ml.

For the inhibition tests, several samples were spiked with the same final concentration of RoV RNA, previously isolated from the RoV suspension, but including different amounts of inhibitory effluent. An aliquot of an effluent sample from the wastewater treatment plant was sterile filtrated through 0.2 μm Minisart NML 16534 filter (Sartorius, Germany). The effluent concentrations tested were 90, 10, 7, 3, 0.7, 0.3 % (*v*/*v*) effluent in milliQ water. Filtered effluent and milliQ water both tested negative for RoV. Samples were stored at −20 °C until use. In order to compare the inhibitory effects of the effluent on the performance of the two assays, rotavirus copies measurements were normalized relative to the measured virus copies in the sample containing 0 % of effluent (inhibitor).

For evaluation of the applicability of the method to environmental waters, different surface water samples were collected. They were sterile filtrated through 0.2 μm Minisart NML 16534 filter (Sartorius, Germany) to remove larger particles, and then they were spiked with the same amount of RoV (990 μl of each sample was spiked with 10 μl of RoV suspension). RNA was then isolated from these spiked samples and applied to RT-qPCR and RT-ddPCR based quantification as described below.

### RNA isolation

Viral RNA was in all cases isolated using the QIAamp Viral RNA Kit (QIAGEN, CA, USA) according to the manufacturer’s instructions (see supplementary method). Samples were eluted with molecular grade RNAse free water (Sigma, MO, USA). A negative control for the extraction procedure, consisting of milliQ water instead of sample, was included in each isolation round. Isolated RNA was stored at −20 °C. The reproducibility of the RNA extraction method and the robustness of the qPCR amplification efficiency were confirmed in a separate assay, in which luciferase control RNA (Promega, WI, USA) was spiked, before RNA extraction, into rotavirus samples serially diluted in wastewater plant’s effluent (Table S1, Electronic Supplementary Material).

### Reverse-transcription real-time PCR

The primers and the minor groove binding (MGB) TaqMan probe used in this study for detecting rotaviruses were adapted from [21] using a non-degenerate probe (Vp2-P: FAM-ATGCGCATGTTATCAAACGCAA-MGB).

The AgPath-ID™ One-Step RT-PCR Kit (Life Technologies, CA, USA) was used for the RT-qPCR reaction. Each sample was applied in triplicate to a final 10 μl reaction volume, with final concentrations of primers and probe of 900 and 250 nM, respectively. At least three repeats of no template control (NTC) were included in each analyzed plate. The RNA was denatured at 95 °C for 5 min and kept on ice prior to its addition to the reaction. Plates were analyzed in a 7900HT Fast Real-Time PCR System (AppliedBiosystems, CA, USA). Thermal cycling conditions were as indicated in the AgPath kit. Data were acquired and analyzed using the SDS 2.4 software (AppliedBiosystems, CA, USA). The threshold was set manually at 0.065 (a level that was above the baseline and sufficiently low to be within the exponential increase region of the amplification curve) and the baseline was set automatically.

### Calibration of RT-qPCR assay

Quantification cycle (Cq) values are output data from RT-qPCR, and numbers of measured copies per microliter of reaction from RT-ddPCR. Additional processing of the data was therefore necessary to compare the results of the two approaches.

To generate a standard for the RT-qPCR-based quantification, a suspension with known concentration of RoV particles (1.1 × 10^11^ particles/ml) estimated by electron microscopy counting using latex bead standards was first diluted to concentration 10^10^ particles/ml. From this suspension RNA was isolated as described above (2.3. RNA isolation). Non diluted RNA (estimated 10^10^ RoV genome copies/ml) and its tenfold serial dilutions in molecular grade RNAse free water, from 10^9^ RoV genome copies/ml to 10^0^ genome copies/ml were used for the creation of the calibration curve (Electronic Supplementary Material, Table S1 and Fig. S1). Each RNA dilution was analyzed in triplicates. The equation of the linear regression was then used to obtain the number of detected targets from the Cq values of the samples [22].

### RT-ddPCR

Reaction mixtures in a final 20 μl volume consisted of 10 μl of 2× One-Step RT-ddPCR Supermix (Bio-Rad, CA, USA), 0.8 μl of 25 mM manganese acetate solution (Bio-Rad, CA, USA), 5.2 μl of mixture of forward and reverse primers, probe and molecular grade RNAse free water and 4 μl of RNA. The final concentrations of primers and probe were the same as for RT-qPCR assays. The RNA was denatured at 95 °C for 5 min and kept on ice prior addition to the reaction. Four microliters of RNA from each sample (or molecular grade RNAse free water for NTCs) was transferred into individual wells on a disposable eight-channel droplet generator cartridge (Bio-Rad, CA, USA). Each oil well was filled with 70 μl of droplet generation oil (Bio-Rad, CA, USA) and the prepared cartridge was then loaded into the QX 100 droplet generator (Bio-Rad, CA, USA).

After droplet generation, the suspension of droplets from each well was transferred by pipetting to a 96-well polypropylene plate (Eppendorf, Germany), heat sealed with foil, and amplified in a conventional calibrated GeneAmp System 9700 thermal cycler (AppliedBiosystems, CA, USA). The thermal cycling conditions consisted of 30 min reverse transcription at 60 °C, 5 min initial denaturation at 95 °C, followed by 45 cycles of a two-step thermal profile of 30 s denaturation at 94 °C and 60 s annealing-elongation at 60 °C at 100 % ramp rate (up and down), and a final 10 min denaturation step at 98 °C. After thermal cycling, plates were transferred to the QX 100 droplet reader (Bio-Rad, CA, USA).

Positive droplets, containing amplification products, were discriminated from negative droplets by applying a fluorescence amplitude threshold in QuantaSoft software (Bio-Rad, CA, USA). The threshold was set manually at the highest point of the negative droplet cluster, as visualized using both the fluorescence amplitude vs. event number and the histogram of events vs. amplitude data streams, on the FAM channel. Data generated by the QX 100 droplet reader were rejected from subsequent analysis if a clog was detected by the QuantaSoft software or if a low number (<10,000) of total droplets was identified per 20 μL PCR. All samples were tested in three replicates with both RT-qPCR and RT-ddPCR assays, except for the samples used to determine the RT-ddPCR dynamic range for which five replicates were used.

An RT-ddPCR reaction was considered positive if at least three droplets (out of 20,000 produced in the reaction) were found positive. A sample was considered positive if all replicate reactions were positive.

## Results and discussion

**Fig. 1:**
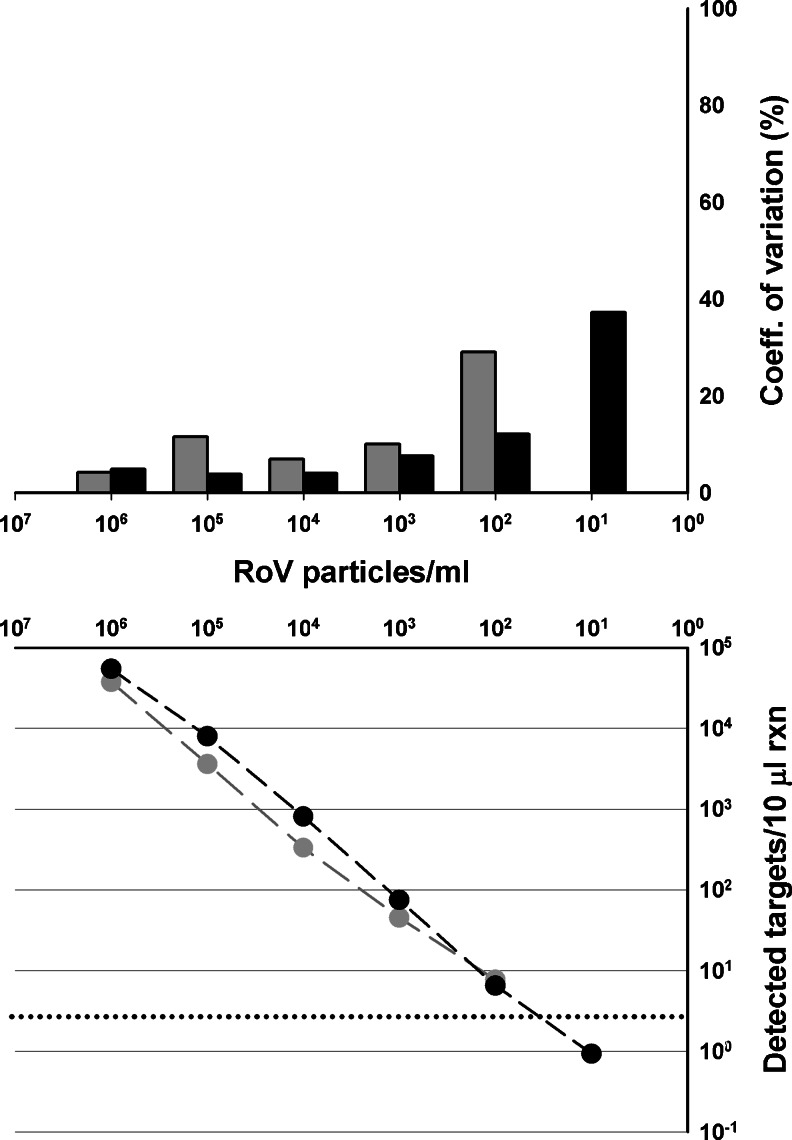
Performances of RT-ddPCR and RT-qPCR in the dynamic range. *Lower panel* target numbers detected per sample, quantified by electron microscopy (RoV particles/ml). *Gray line* RT-qPCR assay; *Black line* RT-ddPCR assay. The *dotted horizontal line* marks the threshold above which the signal is considered positive. Each concentration was applied in three replicates to RT-qPCR and five replicates to RT-ddPCR. The coefficient of variation is shown in the *upper panel. Upper panel* precision of the assays measured by the coefficient of variation (CV) of the measured target RNA copy numbers shown in the *lower panel. Black bars* CV for the RT-ddPCR assay; *gray bars* CV for the RT-qPCR assay. All negative controls were found negative

The sensitivity of the RT-ddPCR assay, assessed on a rotavirus dilution series, is comparable to that of RT-qPCR, with a limit of detection below 10 rotavirus RNA copies/10 μL reaction (Fig. 1 and Table 1). Low-level background signal in some repeats of the samples from 10^2^ to 10^0^ rotavirus particles/ml (Table 1), despite being below the criteria for sample to be considered positive (see “RT-ddPCR”), could be attributed to low-level contamination during preparations of dilution of rotavirus suspensions. Similar observations of low-level background signal in ddPCR reactions were reported before [11] and may be related with the exceptional sensitivity of the method.

**Table 1:** Sensitivity of the RT-ddPCR and RT-qPCR assays

SAMPLE^a^ (RoV particles/ml)	Virus copies added to the reaction^b^ (in 10 μl rxn)	RT-ddPCR			RT-qPCR	
		Number of droplets analyzed (in 20 μl rxn)	Positive droplets (in 20 μl rxn)	Normalized detected targets^c^ (in 10 μl rxn)	Cq values	Detected targets^c^ (in 10 μl of rxn)
10^10^	6.2 × 10^7^	1,677^d^	1,660	/	16.8	41,067,415
		15,021	15,019	98,100	16.7	44,279,568
		13,841	13,802	64,500	16.7	43,015,498
10^9^	6.2 × 10^6^	13,209	13,113	54,100	20.0	4,292,248
		13,729	13,729	10,000,000	20.0	4,307,364
		13,208	13,208	10,000,000	20.0	4,346,225
10^8^	6.2 × 10^5^	14,176	13,996	48,000	23.3	435,978
		15,470	15,450	73,100	23.4	428,256
		12,875	12,872	91,900	23.5	394,917
10^7^	6.2 × 10^4^	13,086	12,961	51,100	27.0	35,842
		14,819	14,757	60,200	26.8	38,875
		14,050	13,975	57,500	26.9	37,320
10^6^	6.2 × 10^3^	13,391	7,569	9,150	30.4	3,381
		13,460	7,616	9,170	30.1	4,078
		14,276	8,052	9,120	30.4	3,386
10^5^	6.2 × 10^2^	13,734	1,015	844	33.8	312
		13,441	1,033	879	33.6	355
		14,284	1,129	905	33.8	324
10^4^	6.2 × 10^1^	13,968	106	83.7	36.5	49
		13,438	101	82.9	36.8	41
		15,072	117	85.6	36.6	46
10^3^	6.2 × 10^0^	15,971	36	24.8	39.1	8
		13,155	8	6.7	38.8	10
		13,103	7	5.9	39.7	5
10^2^	6.2 × 10^−1^	13,054	0	0.0	n.d.	n.d.
		12,595	1	0.9	n.d.	n.d.
		13,446	1	0.8	n.d.	n.d.
10^1^	6.2 × 10^−2^	14,317	1	0.8	n.d.	n.d.
		13,167	1	0.8	n.d.	n.d.
		15,160	0	0.0	n.d.	n.d.
10^0^	6.2 × 10^−3^	14,354	1	0.8	n.d.	n.d.
		13,598	1	0.8	n.d.	n.d.
		13,337	0	0.0	n.d.	n.d.
NK	0	12,801	0	0	n.d.	n.d.
		12,907	0	0	n.d.	n.d.
		14,448	3^e^	/	n.d.	n.d.
NTC	0	12,234	0	0	n.d.	n.d.
		13,676	0	0	n.d.	n.d.
		12,242	0	0	n.d.	n.d.

^a^Concentration of RoV particles per ml of a sample as estimated with the electron microscope (EM)

^b^Expected theoretical RoV genome copies in 10 μl of a reaction calculated from EM data. Values were corrected to account for the dilutions used in RNA extraction and amplification, and assuming no losses in the extraction procedure

^c^Numbers of detected targets in 20 μl RT-ddPCR reactions were normalized to a final reaction volume of 10 μl for easier comparison with RT-qPCR. In the case of RT-qPCR, the numbers of detected targets were calculated using the standard curve shown in Fig. S1

^d^This sample did not meet the minimal number of droplets criterion (10,000 droplets) and was not included in the analysis

^e^Signals obtained from these droplets were recognized as false positives due to the abnormally high fluorescence intensity measured

Positive droplets: number of droplets showing positive signal for RoV. Detected targets (RT-ddPCR): number of target RNA measured following the Poisson law. *Cq* quantification cycle values for RT-qPCR. Detected targets (RT-qPCR): number of target RNA detected by RT-qPCR. *n.d.* not detected (negative reaction).

With the ddPCR instrument used in this study, the PCR reaction mixture is separated into 20,000 droplets, enabling a theoretical dynamic range of approximately five orders of magnitude [11]. To determine the dynamic range of the RT-ddPCR assay precisely, a decimal dilution series of viral RNA (Fig. 1) was analyzed. The correlation coefficient (*R* ^2^) obtained by linear regression analysis showed a good linearity of amplification for both RT-qPCR (*R* ^2^ = 0.9961) and RT-ddPCR (*R* ^2^ = 0.9981) assays, demonstrating a satisfactory dynamic range of at least four orders of magnitude. Interestingly, in the lower part of this dynamic range, RT-ddPCR showed excellent measurement repeatability that was unmatched by RT-qPCR, as indicated by the coefficient of variation of the measured rotavirus RNA copy numbers between replicates (below 15 % for RT-ddPCR between 10^7^ and 10^3^ RoV particles/ml, corresponding to concentrations of 54,400 ± 2,632 and 6 ± 1 rotavirus copies/10 μL reaction) (Fig. 1). This result demonstrates the higher precision and repeatability of RT-ddPCR for quantifying waterborne viruses at the low concentrations present in most samples analyzed routinely for water quality [23]. Such higher precision of digital PCR approach vs. qPCR technology for low target concentration herein proved for RNA targets was previously only demonstrated with DNA targets [16, 17, 24].

Another important advantage observed with ddPCR is that it provides an absolute number of viral RNA copies present in the sample. The concentration of RoV particles in the initial suspension used for the experiments was estimated to be 1.1 × 10^11^ particles/ml based on TEM counting with latex beads. After applying a correction factor taking into account the dilutions used in RNA extraction and amplification, and assuming no losses in the extraction procedure, the amount of RoV targets per 10 μl reaction volume is estimated to be approximately 6.2 × 10^7^ in the 10^10^ RoV particles/ml sample and 6.2 in the 10^3^ RoV particles/ml sample. Considering that electron microscope (EM) estimation for rotavirus concentration was done for the viral particles and that RT-ddPCR and RT-qPCR detect RNA, the EM estimation is in good correlation with the quantification done by both methods (Table 1). Theoretically, the dynamic range of a ddPCR reaction is limited by the number of droplets that are analyzed. With the instrument used for this study, a value of 5.9 average target copies per droplet (118,000 targets per 20 μl of reaction), equivalent to 99.5 % of the droplets hosting target RNA, was defined as the theoretical upper limit that can ensure quantitative data acquisition [17]. Therefore, for the highest concentrations tested (samples containing 10^10^ to 10^8^ RoV particles/ml), the number of rotavirus copies was above the instrument upper range of quantification, near saturation with almost 100 % of the analyzed droplets containing rotavirus cDNA copies (Table 1). At these levels of concentration, despite the presence of rotavirus being detected, the Poisson law can no longer be applied and the concentration of targets cannot be determined. In water samples, viruses are usually less abundant than these limits. In most cases, the concentration of viruses in a sample can be quantified by RT-ddPCR. However, for higher virus loads, samples have to be diluted before measurement with RT-ddPCR. Alternatively, a ddPCR instrument enabling creation of a larger number of droplets would increase the available dynamic range and allow direct quantification of samples with higher levels of virus [10].

RT-ddPCR and RT-qPCR assays were also carried out on spiked environmental samples. The rotavirus copy numbers measured with the two assays were comparable, but lower variability (as determined by the coefficient of variation of the virus copy numbers measured between replicates) was observed with RT-ddPCR, confirming the overall higher precision of this technique relative to RT-qPCR, even when different water matrices are considered (Table 2).

**Table 2:** Analysis of spiked environmental samples showing average numbers of copies detected by RT-ddPCR and RT-qPCR

Water source	pH	Conductivity (μS/cm)	Turbidity (NTU^a^)	RT-ddPCR^b^		RT-qPCR	
				Average	CV^c^ (%)	Average	CV^c^ (%)
Ljubljanica river	8.43	423	0.78	3,173	1.9	1,385	15.1
Gradaščica creek	8.36	464	3.79	2,920	0.6	1,592	4.0
Tivoli pond	8.29	340	26.18	2,043	3.4	1,244	7.1
Spring near Trzin	8.26	549	12.77	3,637	3.2	2,024	3.1
Pšata river	8.31	405	0.11	3,033	1.7	1,632	14.1
Tap water	7.42	497	0.07	3,440	4.1	1,970	5.9
milliQ water	6.21	3	0.00	3,590	1.5	2,131	1.3

Average: average rotavirus RNA copy values (*n* = 3) in a final reaction volume of 10 μl. Remark: all NTC and negative controls were negative

^a^ *NTU* Nephelometric Turbidity Unit

^b^Number of detected targets in 20 μL RT-ddPCR reactions were normalized to a final reaction volume of 10 μl for easier comparison with RT-qPCR

^c^ *CV* Coefficient of variation between replicates (*n* = 3)

**Fig. 2:**
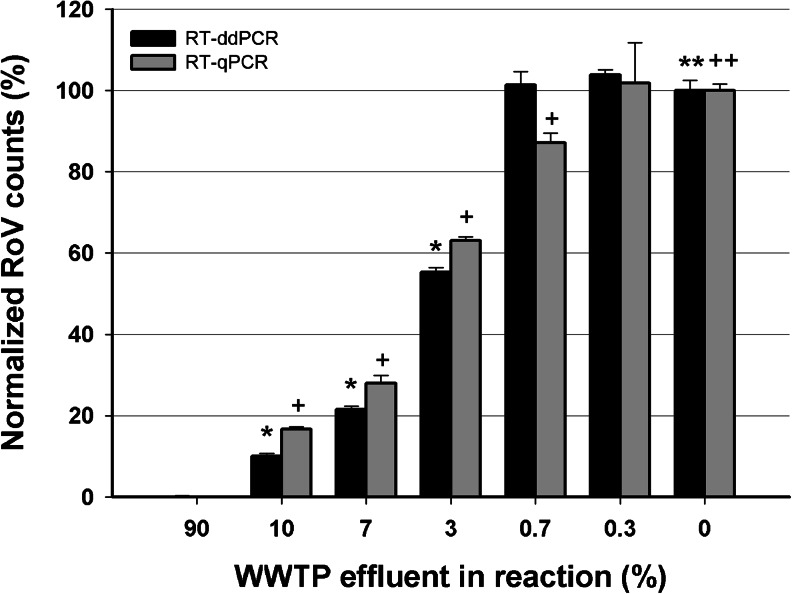
Susceptibility to inhibition of RT-ddPCR and RT-qPCR assays. Samples with different concentrations of effluent from a wastewater treatment plant, diluted in milliQ water, were spiked with the same amount of RoV inoculum. The target RNA copy numbers measured by RT-ddPCR and RT-qPCR were normalized to those measured in 0 % effluent (absence of inhibition). *Error bars* denote the coefficient of variation between three replicates for each measurement. *RoV* Rotavirus. *WWTP* water waste treatment plant * and *+* denote measurements that differ statistically (*t* test, *p* = 0,05) when compared to the control sample without effluent (denoted ** or *++*, for RT-ddPCR or RT-qPCR)

Inhibition is often a problem when detecting or quantifying targets in environmental water samples by qPCR-based methods [8, 9]. We tested the susceptibility to inhibition of both RT-ddPCR and RT-qPCR assays by comparing their ability to quantify a constant amount of rotavirus RNA target in the presence of different concentrations of effluent from a wastewater treatment plant (Fig. 2). In the presence of 90 % effluent, both assays were strongly inhibited, with RT-ddPCR detecting only 1 % of the rotavirus RNA copies and RT-qPCR assay being totally inhibited (no signal). Throughout the dilutions of effluent, the susceptibility of both assays to inhibition was similar down to 0.7 % of effluent, below which the amounts of target measured by RT-ddPCR were comparable to those obtained for the uninhibited control (0 % effluent). At 0.7 % effluent however, RT-qPCR was still significantly inhibited and underestimated the number of rotavirus RNA copies by more than 10 %. Thus RT-ddPCR appears less prone to inhibition, as proposed earlier [17, 18].

## Conclusions

In conclusion, one-step RT-ddPCR is comparable to RT-qPCR in terms of sensitivity, but shows superior quantitative performance and better tolerance to matrix inhibition when applied to RNA virus analysis in water samples. The adaptation of the RT-qPCR assay to a one-step RT-ddPCR assay was straightforward for this particular case. In view of the future adoption of strict quantitative rules [25], the easy conversion of routinely used, validated qPCR assays to ddPCR assays would allow the enforcement laboratories to precisely and absolutely quantify waterborne viruses without the need of calibrant. Assay redesigning and optimization may help solving potentially undesired problems if they would appear, i.e., inability to clearly distinct among negative and positive droplet clusters. An important advantage is that RT-ddPCR performance makes it fully suitable for the quantification of low amounts of viruses expected in water samples. In the most difficult matrices such as the effluent samples from a waste-water treatment plant, RT-ddPCR is still able to quantify viruses in presence of moderate inhibition, unlike RT-qPCR. Therefore, RT-ddPCR offers a straightforward and more accurate quantification approach to respond to the new requirements for microbial water quality. This new concept meets the demand for accurate waterborne virus quantification with a convenient, absolute approach. In addition, the potential of one-step RT-ddPCR may be extended to the quantification and quality control of RNA based reference materials typically used in diagnostics and metrological laboratories.

## Electronic supplementary material

Below is the link to the electronic supplementary material.

ESM 1 (PDF 251 kb)
