# Supplementary material for: One-step RT-droplet digital PCR: a breakthrough in the quantification of waterborne RNA viruses
Source: Anal Bioanal Chem. 2013 Nov 26;406(3):661–7. doi: 10.1007/s00216-013-7476-y (PMC3892107; doi:10.1007/s00216-013-7476-y)
Supplement: Supplementary file 1 — (PDF 251 kb) [file 216_2013_7476_MOESM1_ESM.pdf]

Analytical and Bioanalytical Chemistry

Electronic Supplementary Material

One-step RT-droplet digital PCR: a breakthrough in the quantification of waterborne RNA viruses

Nejc Rački, Dany Morisset, Ion Gutierrez-Aguirre, Maja Ravnikar

Table S1. Evaluation of the performance of the extraction procedure and the amplification using spiked luciferase control RNA

**EFFLUENT SAMPLE** [a] (RoV particles/ml)	**Luciferase control** (Cq)	Average
	21.7	
**10^{10}**	21.5	21.7
	21.9	
	21.7	
**10^{9}**	21.7	21.7
	21.7	
	21.9	
**10^{8}**	21.7	21.7
	21.5	
	21.6	
**10^{7}**	21.8	21.8
	21.9	
	21.7	
**10^{6}**	21.6	21.7
	21.8	
	21.9	
**10^{5}**	21.9	21.8
	21.8	
	22.6	
**10^{4}**	22.6	22.2
	21.4	
	21.8	
**10^{3}**	21.8	21.7
	21.5	
	21.9	
**10^{2}**	22.0	22.0
	22.2	
	22.1	
**10^{1}**	22.2	22.1
	21.9	
	22.7	
**10^{0}**	22.8	22.6
	22.4	
	n.d.	
**NTC**	n.d.	n.d.
	n.d.	

[a] Concentration of RoV particles per ml of the spiked effluent sample estimated with the electron microscope (EM)

[b] Negative control of isolation (NK), as well as the highly diluted samples are positive for rotavirus due to a residual presence of the virus in the effluent that was used for spiking.

Supplementary method: extraction performance evaluation

A RoV suspension (1.1 x 10^{11} rotavirus particles/ml) was first diluted to 10^{10} rotavirus particles/ml, in (waste water) effluent. From this concentration further 10-fold serial dilutions in effluent were prepared all the way to 10^0 rotavirus particles/ml. 2 ng of luciferase RNA were added in each dilution, then RNA was extracted (see 2.3 RNA isolation) and each extract was applied to luciferase specific assay. The Cq of luciferase were constant, Cq 21-22, (Table S1), indicating that the extraction and amplification are unaffected, even in samples diluted in wastewater treatment plant effluent. The obtained luciferase Cq, were in the same range as when luciferase was spiked in buffer or RNAase free water before extraction (data not shown). This indicates that the extraction and amplification are not affected by the inhibitors that are introduced by the effluent in the sample.

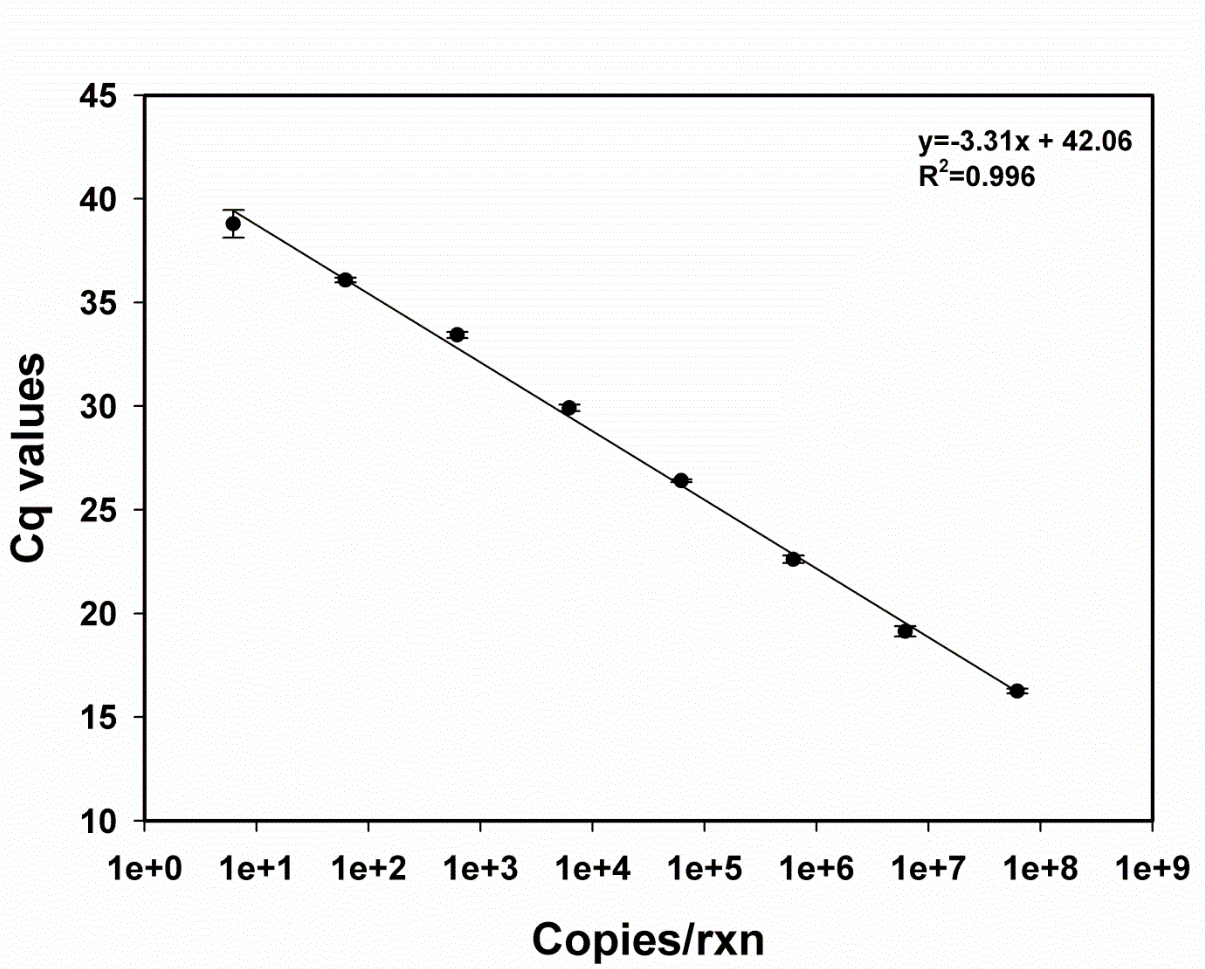

Fig. S1. Calibration curve for the RT-qPCR assay

Correlation between Cq values and RoV copies per reaction estimated using dilutions of RNA purified from a known concentration rotavirus suspension quantified by counting under electron microscope using latex beads as standard. In the top right corner the equation and correlation coefficient (R^2) for the calibration curve are given. Plotted Cq values are average values from triplicate measurements. Error bars denote the standard deviation from triplicate measurements.
